# Emerging Targeted Therapeutics for Genetic Subtypes of Parkinsonism

**DOI:** 10.1007/s13311-020-00920-8

**Published:** 2020-09-10

**Authors:** Susanne A. Schneider, Baccara Hizli, Roy N. Alcalay

**Affiliations:** 1 Department of Neurology, Ludwig-Maximilians-University of München, Marchioninistr. 15, 81377 Munich, Germany; 2 Department of Neurology, Columbia University Irving Medical Center, New York, NY USA

**Keywords:** Genetic Parkinson’s disease, SNCA, GBA, LRRK2, kinase inhibitor, small molecule compounds, Venglustat, ambroxol, clinical trial, TORC1 inhibitor

## Abstract

In recent years, a precision medicine approach, which customizes medical treatments based on patients’ individual profiles and incorporates variability in genes, the environment, and lifestyle, has transformed medical care in numerous medical fields, most notably oncology. Applying a similar approach to Parkinson’s disease (PD) may promote the development of disease-modifying agents that could help slow progression or possibly even avert disease development in a subset of at-risk individuals. The urgent need for such trials partially stems from the negative results of clinical trials where interventions treat all PD patients as a single homogenous group. Here, we review the current obstacles towards the development of precision interventions in PD. We also review and discuss the clinical trials that target genetic forms of PD, i.e., *GBA*-associated and *LRRK2*-associated PD.

**Electronic supplementary material:**

The online version of this article (10.1007/s13311-020-00920-8) contains supplementary material, which is available to authorized users.

Parkinson’s disease (PD) is the second most common neurodegenerative disorder, affecting more than 6 million people worldwide [1]. Numerous drugs for the treatment of motor and non-motor symptoms of PD are available on the market. While these drugs improve motor symptoms and quality of life of people with PD, there is no evidence that any of these interventions modify the progression of PD pathology. There are no FDA-approved interventions to slow down PD progression even though there have been multiple clinical trials for treatments that would modify the PD pathophysiological process. One potential explanation of this challenge is that PD is not a single patho-biological process. Consequentially, a tailored approach, where interventions are selected by the patients’ genotype or other biomarkers (as a measure of a biological process associated with a disease state), is required. In parallel, our knowledge of genetic risks has expanded significantly in the past decade and makes a precision medicine approach in PD very timely. Here, we review the obstacles towards the completion of clinical trials in PD along with the efforts to overcome these challenges. We outline the currently available data on precision medicine clinical trials, focusing primarily on *alpha synuclein* (*SNCA*), *glucocerebrosidase* (*GBA*), and *Leucine-Rich Repeat Kinase 2* (*LRRK2*).

## Precision Medicine

Precision medicine aims to tailor treatment in a personalized manner, for the right person at the right time. To achieve this, it uses diagnostic tools to identify specific biomarkers, often genetic, to help assess which medical treatments will be best for each patient [2]. Thus, in addition to the mission of providing personalized medicine, the term precision medicine also conveys the concept that genomics and other emerging biodata sciences will improve medicine’s clinically defined nosology [3].

The hypothesis underlying precision medicine is that diseases previously treated as a single condition are actually biologically different, and that treatment should be tailored based on the biological signature of the individual (stratified medicine [3]). A successful example is oncology, where precision medicine led to the identification of effective therapies based on tumor profiling and a better understanding of how tumors become resistant to cancer therapy (https://www.cancer.gov/research/areas/treatment/pmi-oncology). In PD, precision trials are ongoing for genetic forms of PD.

## Genetic Underpinnings of Parkinson’s Disease

**Fig. 1:**
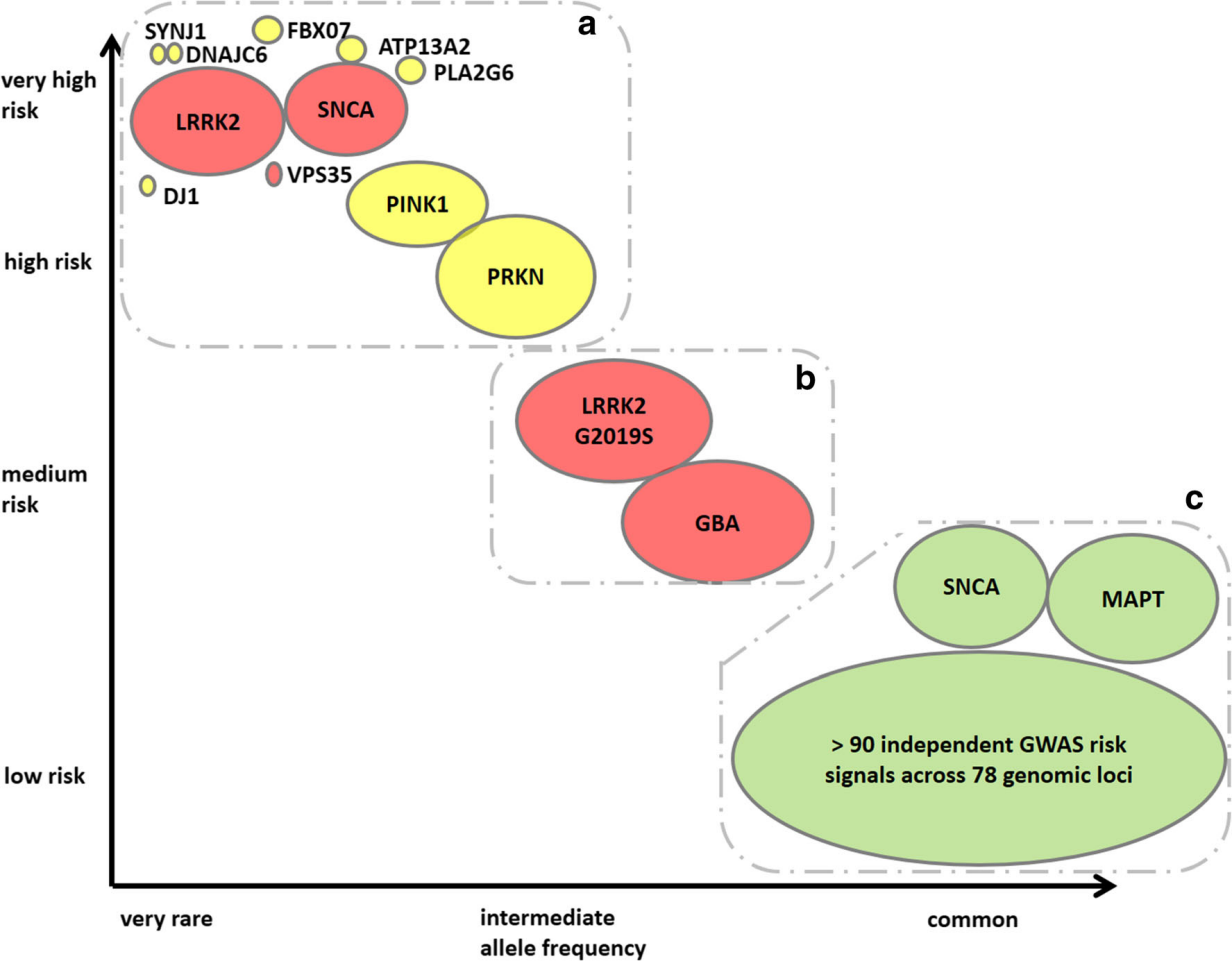
Genetic architecture of Parkinson’s disease, modified from [4] and [5], showing the continuum of variants of different effect strengths and allele frequencies. The size of the bubbles roughly corresponds to population allele frequencies. Colors symbolize modes of inheritance: dominant (red), recessive (yellow), risk loci (green). The genetic risks for PD can be roughly divided into three groups (dotted areas; see main text for more details). The group of common low-risk genes (lower right corner) includes more than 90 putative independent genome-wide significant signals identified in a GWAS meta-analysis based on more than 13,000 patients and 95,000 controls [6]. Similar to *LRRK2* and *SNCA*, some mutations in *VPS13C* and *GCH1* are likely causal or confer high risk for PD [6]

In recent years, our understanding of the genetics of PD has expanded significantly. In addition to over a dozen Mendelian loci (Fig. 1), multiple PD genetic risk factors have been identified. These risk factors are mostly associated with a small increase in risk of PD (e.g. up to ~1.5-fold) but occur as commonly as 40% in the general population [5]. It is thought that the overall heritable component of disease is about 30% (5–10% due to monogenic causes and around 22% driven by common variants identified by genome-wide association studies) [6–8]. A prevailing hypothesis is that many of the identified genes and risk factors map into two closely related and overlapping cellular pathways: mitochondrial metabolism and autophagy. In addition, other pathways such as endosomal trafficking, synaptic transmission and immune response pathways have been identified to play a role in the pathogenesis of PD. [7, 9] While these biological pathways have been linked to PD via genetic studies, it is very likely that dysfunction in these same pathways due to environmental exposures could also lead to PD. Figure 1 describes the genetic architecture of PD.

The genetic risks for PD can be roughly divided into three groups, based on their frequency in the general population and the PD risk they convey. Accordingly, clinical trial designs for each one of these groups may also differ.

1. **Group A** includes most of the Mendelian genes linked to PD (shown in the left upper part of Fig. 1). These mutations convey a major risk factor for PD, but are extremely rare. In most cases, mutations are present in too few patients for a standard clinical trial design (e.g., *PINK1* or *PARK7*). Intervention on such mutations would require a different study design such as patient-customized therapy. A recent report of patient-customized intervention in a child with neuronal ceroid lipofuscinosis 7 (CLN7, a form of Batten’s disease) demonstrates the potential of this type of study [10]. Among all of these rare PD mutations, mutations in *SNCA* and *Parkin* may be more common. Indeed, if a sufficient amount of *SNCA* mutation carriers are identified, there may be promising interventions in this patient population group including silencers of the *SNCA* gene (e.g., antisense oligonucleotides (ASOs), viral-mediated delivery of siRNA, and miRNA. Of these, ASOs have recently shown favorable results in patients with spinal muscular atrophy [11] and Huntington’s disease [12]. As described below, these treatments may also be useful for a broader range of PD patients.
2. **Group C** includes variants which convey a very low increased risk for PD (shown in green on the right in Fig. 1), but may be present in a large percentage of people with PD. These variants may be common enough that intervention on the biological pathway of one of these variants may not require pre-screening clinical trial candidates, but instead result in stratifying the participants based on the presence of the variant. For example, a major pathway highlighted by GWAS analysis in large PD cohorts is the adaptive immune system. This was first identified through a human leukocyte antigen association but is now increasingly recognized as a major determinant of risk across the genome [13]. We are not aware of any current clinical trials that focus on a Group C gene for PD. However, ASOs (developed by Ionis) targeting tau (*MAPT*) are being studied in a phase I clinical trial for Alzheimer’s disease. In addition, retroactive analyses of published clinical trials may identify responders versus non-responders based on variant status. It is possible that unsuccessful clinical trials for disease-modifying agents would have been successful in a select group of people with PD.
3. **Group B** includes variants that are shown in the middle of Fig. 1. They are deleterious enough (associated with a high enough risk for PD) to warrant an intervention, but also common enough to make clinical trials targeting mutation carriers with a reasonably high number of participants feasible. Currently, this group includes variants in *GBA* and *LRRK2.* Given tremendous efforts to identify research cohorts, (e.g., by the Michael J. Fox Foundation-funded PPMI studies) our knowledge of the PD phenotype and the risk for PD among these mutation carriers has improved [14, 15].

The differences among the groups (A/B/C) are derived from the frequency of the variant in the population (*x*-axis), and the risk for PD associated with the variant (*y*-axis). The relationship between frequency and PD risk can guide the study design and who will benefit from particular interventions. Specifically, mutations in group A are rare, and interventions on these targets may not benefit all PD cases. One example of this is homozygous carriers of *PRKN* mutations. We are not aware of such studies in group A in PD but an example from the dementia field would be for intra-cisternal PR006 administration of the progranulin protein (*PGRN*) in carriers of the progranulin gene mutation (https://clinicaltrials.ucsf.edu/trial/NCT04408625). This type of intervention would not be for all patients with dementia but would only include mutation carriers. The study design would have to include genotype as an inclusion criterion and a large number of PD cases would have to be genotyped to identify a small number of participants. Variants in group C are common and intervention on them may be beneficial for all PD cases. An example would be interventions on alpha-synuclein (α-syn) in PD. These studies do not require prescreening for genetic variants prior to study participation.

## Current Research and Clinical Trials for Genetic Forms of PD

### *SNCA-*Associated Parkinsonism: Active and Passive Immunization

**Fig. 2:**
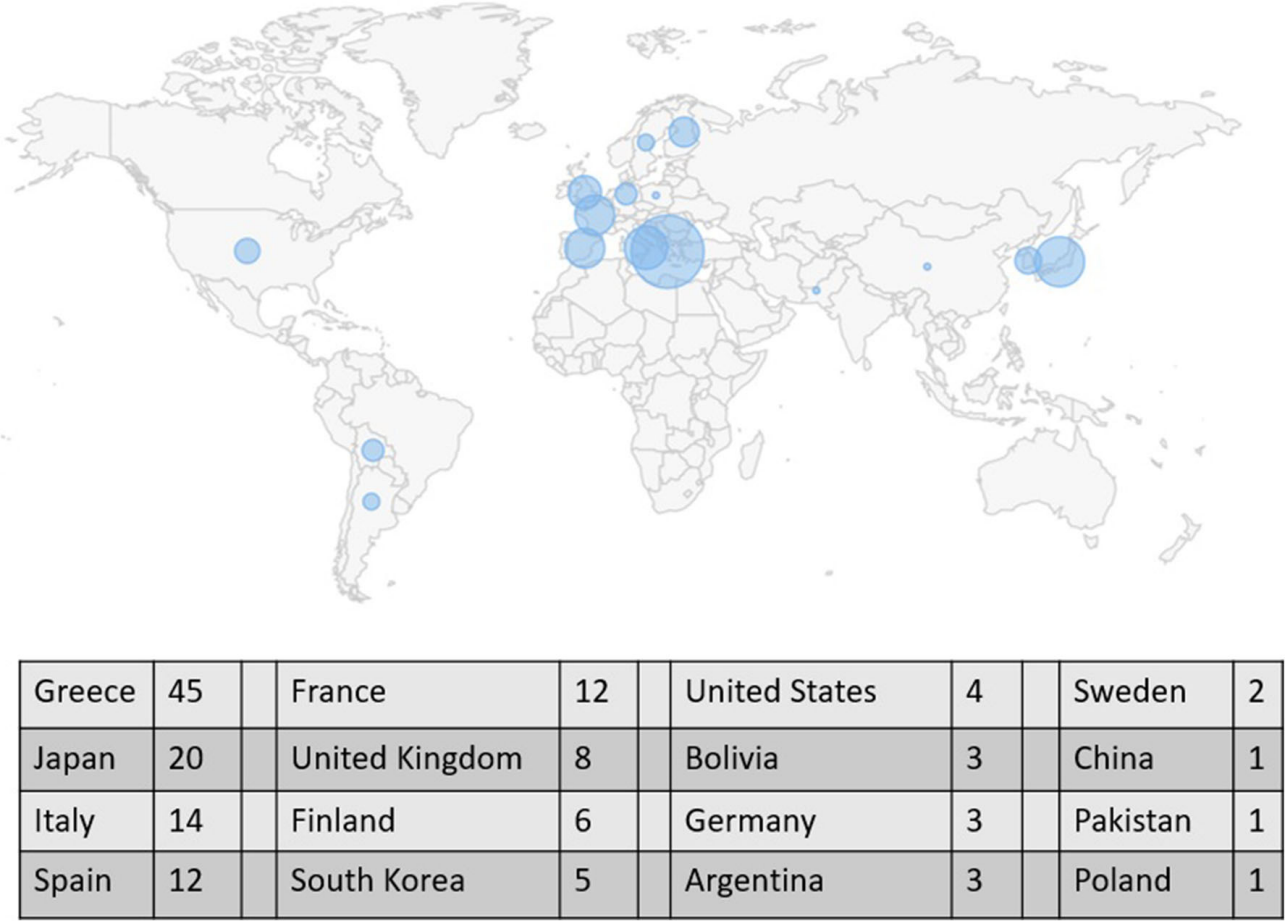
World map of *SNCA*-associated Parkinsonism. A total of 140 cases have been reported in the literature. Circles reflect frequency per region. Data and image were retrieved from the MDSGene Website [21]

Discovered in 1997, *SNCA*, which encodes the protein product α-syn, was the first cloned gene for PD. Soon after, it was discovered that α-syn is a key component of Lewy bodies which are considered to be a pathological hallmark of PD. Therefore, the link between α-syn and PD seems clear and extends beyond the genetic cases caused by *SNCA* mutations. The physiological state of α-syn is an intrinsically disordered monomer or helically folded tetramere that has toxic effects in its oligomeric form. The protein is degraded mainly by the autophagic system. Pathogenic missense mutations (A53T [16], A30P [17], E46K [18] and H50Q [19] and G51D [20] [PARK1] which leads to misfolding and promotes apoptosis) and changes in gene dosage (duplications, triplications [PARK4] which leads to an excess and aggregation of α-syn) are associated with PD (Fig. 2). The prevailing hypothesis is that excess α-syn is pathogenic and that mutations in the *SNCA* gene lead to gain-of-function. Indeed, the clinical phenotype of mutation carriers has been associated with early-onset rapid motor progression and frequent dementia; the phenotype may also overlap with multiple system atrophy (MSA) or dementia with Lewy bodies (DLB). Following the dosage effect, the disease may be more rapidly progressive in triplication carriers versus duplication carriers.

Multiple clinical trials are targeting alpha-synuclein. While *SNCA* mutation carriers would naturally benefit from such interventions, given the difficulty recruiting large enough cohorts of these carriers, none of the trials focuses solely on this population. Since α-syn deposits are universally present in PD and MSA, the trials include both PD and MSA patients. While a very attractive drug target, one of the challenges of the field is that there are no α-syn tracers available. Developing α-syn tracers would help advance the field as they may serve as biomarkers, specifically for target engagement.

Multiple mechanisms of actions are currently being studied for α-syn, including immunotherapies, gene therapies, and small molecules (Table 1).

**Table 1:** Compounds under development targeting alpha-synuclein

Mechanism of action	Compound	Sponsor	Phase/status	*N* of patients	Recruited participants	RCT no.
Active immunization	PD01A, PD03A	AFFITOPE	Phase 1, completed	36	PD	NCT02267434
Passive immunization	BIIB054 (SPARK Study)	Biogen	Phase 2, active, not recruiting, estimated completion 06-2021	311	PD	NCT03318523
Passive immunization	Prasinezumab, PRX002/RO7046015 (PASADENA Study)	Prothena/Roche	Phase 2, active, not recruiting, estimated completion 02-2021	316	PD	NCT03100149
Passive immunization	MEDI1341	AstraZeneca and Takeda	Phase 1, recruiting, estimated completion 01-2021	48	healthy	NCT03272165
Passive immunization	Rec47	n/a	Preclinical			
α-syn aggregation inhibition	NPT200-11, NPT100-18A	Neuropore	Phase 1, completed	55	Healthy	NCT02606682
α-syn aggregation inhibition	NPT-088	Proclara (formerly Neurophage)	†			n/a
α-syn aggregation inhibition	Anle138b [*]	n/a	Preclinical, a clinical trial in PD is expected to begin end of 2020			n/a
α-syn aggregation inhibition	CLR01 [*]	n/a	Preclinical			n/a
α-syn aggregation inhibition	PBT434 [*]	Alterity Therapeutics Limited	Phase 1	70	MSA	n.k.
α-syn aggregation inhibition	VX-765[*]	n/a	Preclinical			n/a
α-syn degradation enhancers	Rapamycin (Sirolimus)[*]	NYU Langone Health, NINDS	Phase 2, recruiting, estimated completion 08-2021	56	MSA	NCT03589976
α-syn degradation enhancers	Micro-RNA-101 [*]	n/a	Preclinical			n/a
α-syn degradation enhancers	TLR4-agonist MPLA [*]	n/a	Preclinical			n/a
Nanobodies	VH14*PEST	n/a	Preclinical			n/a
Epigenome editing	n/a	Duke University/Seelos Therapeutics	Phase 1			n/a

n/a = not applicable; n.k. = not known

*Compounds primarily being tested in MSA

^†^Trial completed in individuals with probable Alzheimer’s, may be applicable to synucleinopathies

### Immunotherapies

Immunotherapies include active immunization (i.e., a vaccination which triggers the immune system to generate an immune reaction, including antibodies, against α-syn), and passive immunization (i.e., administration of antibodies directed against different domains of α-syn). Since it is estimated that antibodies would be most effective against extracellular proteins, these immunotherapies are designed to reduce the load of extracellular α-syn, stop the spread of α-syn pathology in the brain, and/or neutralize its toxic effects. While active vaccinations are generally associated with a higher potential risk of side effects, so far no relevant safety and tolerability issues have occurred in the current α-syn studies. However, active vaccination strategies (such as AFFITOPE) have only been tested in phase 1 human trials.

Of the passive vaccinations, two compounds have reached the clinical phase 2 study level and are currently tested in patients with early PD: PRX002 (PASADENA study, sponsored by Prothena) and BIIB054 (SPARK study, sponsored by Biogen). The first antibody, prasinezumab (PRX002/ RO7046015), binds to the C-terminus of α-syn. A study in healthy volunteers [22] and in mild-to-moderate PD patients [23] demonstrated a favorable safety and tolerability profile which led to the international PASADENA study. A total of 68 centers in the USA and Europe (i.e., Austria France, Germany, and Spain) participate in this 52-week trial. Completion is estimated for early 2021.

The second α-syn antibody, BIIB054, binds to the N-terminus of α-syn, to residues 1–10 [24]. BIIB054 is highly selective for aggregated forms of α-syn with at least an 800-fold higher apparent affinity for fibrillar versus monomeric recombinant α-syn and it has a strong preference for human PD brain tissue. Efficacy studies in three different mouse models with intracerebrally inoculated preformed α-syn fibrils showed that BIIB054 treatment attenuated the spreading of α-syn pathology, rescued motor impairment, and reduced the loss of dopamine transporter density in dopaminergic terminals in the striatum. This led Biogen to initiate the international SPARK study which consists of monthly IV infusions (at 250 mg, 1250 mg, or 3500 mg) over 52 weeks. Another compound in the pipeline for passive immunization is MEDI1341 [25] (phase 1).

### Small Molecules

Another approach that takes a different route of action is small molecules which penetrate the blood–brain barrier and modify α-syn biology. These include inhibition of α-syn misfolding and aggregation and reduction of cortical pathology (NPT200-11 [26], currently in phase 1). Another small molecule mechanism utilized is targeting misfolded or aggregated proteins (NPT088, a second-generation immunoglobulin-general amyloid interaction motif (Ig-GAIM) molecule, which is being developed by the company Neurophage). In the case of an α-syn overexpression-based PD rat model, VH14*PEST (a nanobody which targets the non-amyloid component region) showed maintenance of striatal dopaminergic tone and modest behavioral improvement [27]. Nanobodies are genetically engineered fragments of antibodies which can modulate monomeric concentrations of target proteins.

### Gene Therapy

Another potential strategy is gene therapy, namely the CRISPR-dCas9 epigenome-editing approach which manipulates endogenous *SNCA* levels. Researchers at Duke University (now further developed by Seelos Therapeutics) designed an all-in-one lentiviral which harbors gRNA, dCas9 nuclease, and the catalytic domain DNMT3A to target specific hypomethylated CpG islands in the *SNCA* intron 1 region. This led to the downregulation of *SNCA* mRNA and protein levels and phenotypic perturbations in pluripotent stem cells of a *SNCA* triplication carrier [28].

Given PD’s shared pathophysiological basis with MSA (i.e., both are synucleinopathies), it will be interesting to follow current developments in MSA as treatments may also be effective in PD. α-syn aggregation inhibitors (e.g., Anle138b, CLR01, PBT-434, and VX-765), passive immunization (rec7), and α-syn degradation enhancers (e.g., rapamycin/sirolimus) have been investigated for MSA; for a review, see Heras-Garvin and Stefanova [29].

### *LRRK2*-Associated Parkinsonism: Kinase Inhibitors Are a Promising Target

More than 700 *LRRK2* cases have been reported in the literature. The International *LRRK2* Consortium study estimated that the most common mutation in *LRRK2*, G2019S, accounts for 1% of sporadic and 4% of familial PD patients [30]. Mutations in *LRRK2* are more common in certain ethnicities. North African Arabs (36% in familial, 39% in sporadic) and Ashkenazi Jews (28% in familial, 10% in sporadic) have the highest frequencies [30]. The G2019S mutation is common in Whites, while G2385R and R1628P are the most frequent variants in Asian patients (detected in around 5–10% of Asian PD patients) [31]. Penetrance of *LRRK2* is age dependent and estimations in the general population are variable, ranging between 30% and 74% [32]. There are multiple reasons for this wide range of penetrance estimates, including differences in study design, the investigation of different populations, and the heterogeneity of *LRRK2* presentation. Regardless, the wide range of penetrance estimation makes trial design for preventative interventions extremely difficult.

While the mechanisms by which *LRRK2* mutations cause PD have not been completely disentangled, there is increasing evidence of increased LRRK2 kinase function in mutation carriers with PD. The G2019S mutation, for example, results in a direct two-to-threefold increase in kinase activity [33, 34].The potential gain-of-function effect is an attractive target for treatment because inhibition is easier to achieve than improvement of reduced protein activity (as in *GBA*). Furthermore, kinase inhibitors are widely used in oncology and the PD field can benefit from advancements in other fields. Since the first generation of LRRK2 inhibitors, newer compounds have progressively improved in potency, selectivity, and brain penetrance. However, efficacy and safety remain a concern. This is mainly due to the fact that other tissues, particularly the kidney, lung, and a subtype of peripheral immune cells, robustly express LRRK2. For example, the kidney has a ~6.2-fold higher expression of LRRK2 compared to the brain [34]. The expression of *LRRK2* in other tissues is a potential source of peripheral side effects, which can include the development of lamelar bodies and impaired autophagy-lysosomal function induced by LRRK2 inhibitors [35–37]. More recent data, however, suggest compounds that only partially downregulate LRRK2 levels or kinase activity, i.e., by 50% or less, are unlikely to produce major side effects related to on-target safety [38] and lipid droplets in lamelar bodies are absorbed after the drug is stopped.

One alternative to avoid systemic toxicity is to find a way to specifically modify LRRK2 activity in the brain without modifying activity peripherally. New drug modalities that keep peripheral side effects to a minimum include PROTACs (PROeolysis TArgeting Chimera) which selectively induce intracellular proteolysis via the ubiquitin–proteasome system of the mutant without affecting WT-LRRK2 [39]. However, due to their molecular size, PROTACs show poor permeability across cell membranes which might affect brain penetration [39]. Another alternative to avoid systemic side effects is currently tested by Biogen, who launched a phase 1 antisense oligonucleotide (ASO) trial. Participants will receive a single intrathecal injection of the compound BIIB094, an ASO, on multiple days. Recently, ASOs have been approved by the FDA for spinal muscular atrophy and are being tested for Huntington’s disease and non-neurological disorders such as cancer [40, 41]. ASOs reduce the expression of a mutated gene by binding to target mRNAs and either block the translation of the abnormal protein or induce its degradation [41]. ASOs can also promote splicing around mutations. For disorders caused by toxic gain-of-function mutations such as *LRRK2*, further investigation regarding ASOs is warranted. In a preclinical study, administration of *LRRK2* ASOs to the brains of mice reduced LRRK2 protein levels and fibril-induced α-syn inclusions [42]; data from humans are not yet available.

Several structurally different LRRK2 inhibitors from Genentech, GSK, Merck, and Pfizer are in the pipeline (Table 2) [43]. The compounds developed by Denali are already in clinical trials and a phase 1b trial in healthy individuals has been completed which included pulmonary and renal safety parameters. The company is advancing DNL201 (GNE-7915) to a phase 1b safety and biomarker study in *LRRK2*-linked PD and iPD (idiopathic PD). Twenty-nine mild to moderately affected PD patients with or without *LRRK2* mutation were randomized to either low dose DNL201, high dose DNL201, or placebo in this 28-day randomized placebo-controlled trial. In January 2020, Denali announced that both doses achieved more than 50% inhibition of pS935 LRRK2 and pRab10 phosphorylation in blood, and improved the lysosomal biomarker BMP in urine (by 20% or 60% at the low and high dose) (https://denalitherapeutics.gcs-web.com/node/7361/pdf [online]). The same company also is testing another compound, DNL151. In a trial in 150 healthy volunteers, safety and biomarker goals were met (https://denalitherapeutics.gcs-web.com/node/7361/pdf [online]). The compound is now being tested in PD patients with recruitment in Belgium, the Netherlands, and the UK; however, the trial is delayed because of the COVID-19 pandemic. According to the press release, Denali intends to select either DNL201 or DNL151 in mid-2020 to advance into phase 2/3 clinical trials in patients with Parkinson’s disease (https://denalitherapeutics.gcs-web.com/node/7361/pdf [online]). To facilitate recruitment, a “direct-to-consumer” approach for testing and counseling will be available [44]—a strategy that proved successful in genetic testing with the PPMI initiative. Most recently, Denali has announced a strategic collaboration with Centogene, a gene diagnostic lab, to enhance recruitment [45]. Pharmaceutical companies and academic centers are also conducting observational studies to identify best endpoints related to the *LRRK2* mutation, e.g. typical neurocognitive abnormalities (https://clinicaltrials.gov/ct2/show/NCT01424475?term=lrrk2&draw=2&rank=8).

**Table 2:** *LRRK2*-targeted treatments including LRRK2 inhibitors and antisense oligomeres under development for PD

LRRK2						
Compound	DNL201	DNL151	No public data	No public data	No public data	BIIB094
Sponsor	Denali	Denali	GSK	Pfizer	Genetech	Biogen
RCT no.	NCT03710707	NCT04056689				NCT03976349
Mechanism	LRRK2 inhibition	LRRK2 inhibition	LRRK2 inhibition	LRRK2 inhibition	LRRK2 inhibition	Antisense oligonucleotide (ASO)
Status	Completed	Delayed because of COVID19	Planned	Under development	Under development	Ongoing, estimated completion end of 2022
Phase	Phase 1b	Phase 1b	n/a	n/a	n/a	Phase 1
Design	Multicenter, randomized, placebo-controlled	Multicenter	n/a	n/a	n/a	International, multicenter, placebo-controlled
Total *N* of patients	29	34	n/a	n/a	n/a	62
*LRRK2*-PD	√	√	n/a	n/a	n/a	√
Idiopathic PD	√	√	n/a	n/a	n/a	√
Age	30–75		n/a	n/a	n/a	35–80
Duration	28 days	28 days	n/a	n/a	n/a	n.d.
Doses tested	Low/high	Three doses	n/a	n/a	n/a	Single- and multiple-ascending-dose

#### Deep Brain Stimulation for *LRRK2*-Associated PD

Interestingly, a different level of benefit was noted after deep brain stimulation surgery in *LRRK2*-associated PD. The outcome was better in patients with the most common *LRRK2* mutation, p.G2019S, compared to the poor response seen in patients with the rarer p.R1441G mutation. However, overall numbers remain small. The overall benefit was compromised by the rapid progression of cognitive and neuropsychiatric symptoms [46].

#### Parkinsonism Associated with *GBA* Mutations

Clinical trials that target the glucocerebrosidase (*GBA*) pathway are probably in the most advanced stages towards precision medicine in PD. This is a result of the relatively high frequency of *GBA* mutations in PD and the availability of basic science data collected about *GBA* mutations from Gaucher disease research. *GBA* mutations are a common risk factor for PD and are present in 7–10% of PD patients worldwide. Among Ashkenazi Jews, around 20% of PD patients carry a *GBA* mutation [47]. A high prevalence has also been reported in the Netherlands, where 15.5% of PD patients carry a *GBA* mutation [48].

Homozygous *GBA* mutations cause Gaucher disease (GD), the most common autosomal recessive lysosomal storage disease, with an estimated annual incidence of 1/60,000 and an estimated carrier frequency [49] of 0.7 to 0.8% in the general population. The clinical presentation of GD can be divided into systemic and neurological manifestations. The former, present in all forms of GD, includes hepatosplenomegaly, painful skeletal disorders, and pancytopenia. The latter is present in the more severe types of GD, GD- type 2, and GD-type 3 [50]. More than 300 mutations in *GBA* have been reported [51], and they are traditionally divided based on the GD phenotype they are associated with. Mild mutations (e.g., the N370S mutation) are associated with type 1 GD, while severe mutations (e.g., the L444P mutation) are associated with type 2 or type 3 GD. Some ethnicities show higher mutation rates; specifically, in the Ashkenazi Jewish (AJ) population, the annual incidence is 1/1000 and carrier frequencies are as high as 6% in all AJ. Both GD patients and healthy heterozygous carriers are at increased risk for PD [52]. About 10% of *GBA* mutation carriers will develop PD and studies suggest that penetrance is age dependent [53]. As in LRRK2, there is incomplete penetrance of PD among carriers and a wide range of penetrance estimates.

Parkinsonism in *GBA* heterozygotes may be indistinguishable from iPD. However, they may have an earlier age at onset, more prevalent cognitive impairment, and may not respond to levodopa as well as iPD patients [54, 55]. *GBA* mutations are also associated with other alpha-synucleinopathies, including dementia with Lewy bodies [56] (pathologically confirmed) and, in some studies, with MSA [57–61]. In contrast, there is no association with essential tremor or Alzheimer’s disease.

Pathologically, the brains of patients with heterozygous *GBA* mutations strongly resemble those from patients with iPD. However, there is also widespread cortical Lewy body involvement in *GBA* mutation carriers [55, 61]. A few studies showed a reciprocal relationship between levels of glucocerebrosidase (GCase; the enzyme encoded by *GBA*) and levels of the aggregate-prone protein α-syn [62]. Notably, iPD patients also have reduced GCase activity (about 33% decrease) in the substantia nigra and cerebellum, making treatments that target *GBA* relevant for iPD and patients with PD dementia as well [11]. 

The GBA-encoded protein, glucocerebrosidase, is a lysosomal enzyme which plays a role in the breakdown of glucocerebroside and glucosylsphingosine into glucose and ceramide or sphingosine respectively. In GD, there is a lysosomal buildup of the substrate glucocerebroside in the reticulo-endothelial system with reduced clearance. Gaucher disease manifestations are a result of this diminished glucocerebrosidase (GCase) activity. FDA-approved interventions for GD include enzyme replacement therapy (ERT) and substrate reduction therapy (SRT), which are inhibitors of the glucosylceramide synthase enzyme. None of the currently approved drugs penetrate the blood–brain barrier, but theoretically similar interventions that penetrate the blood–brain barrier may modify the PD phenotype.

**Fig. 3:**
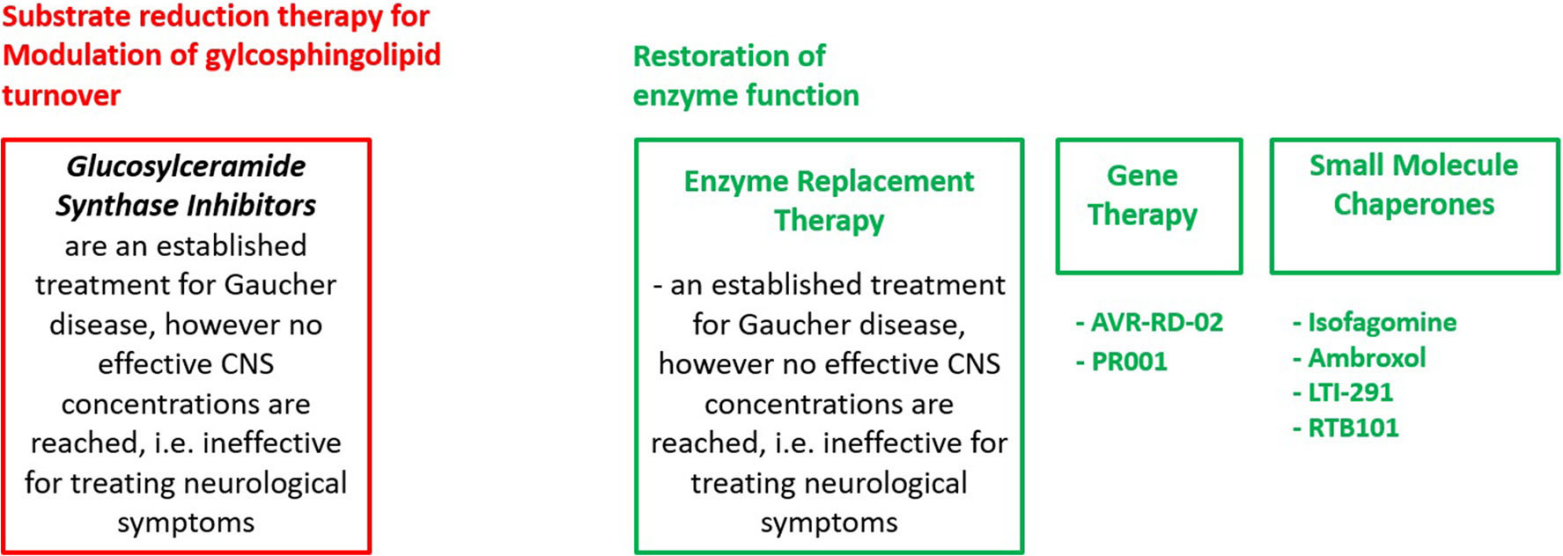
Treatment approaches for *GBA*-associated PD include the modulation of glycosphingolipid turnover and restoration of enzyme function

While the PD field can benefit from decades of GD research, the underlying mechanisms of how *GBA* leads to the development of PD are not fully understood. One hypothesis is that there is a self-propagating bidirectional feedback loop between GCase and α-syn. Loss of GCase activity causes α-syn accumulation and oligomerization, resulting in neurotoxicity through aggregate-dependent mechanisms [63]. Furthermore, elevated α-syn inhibits lysosomal maturation and normal GCase activity while hindering GCase transport from the endoplasmic reticulum to the lysosome. All of these mechanisms continue over time until the threshold for neurodegeneration is reached [63]. Based on this, targeted treatments can take a variety of approaches including modulation of glycosphingolipid turnover and restoration of enzyme function (Fig. 3, Table 3).

**Table 3:** *GBA*-targeting treatments for PD in the clinical phase that aim to modulate glycosphingolipid turnover and restore enzyme function

GBA	MOVES-PD study part 1	MOVES-PD study part 2	AiM-PD			
Compound	Venglustat (GZ/SAR402671)		Ambroxol	RTB101	LTI-291	PR001
Administration	Oral		Oral	Oral		Injections
Sponsor	Sanofi		UCL and Cure PD Trust	Restorbio	LTI/Allergan	Prevail
RCT no.	NCT02906020		NCT02941822		(NL7061; NTR7299) ^a^	
Mechanism	Glucosylceramide synthase inhibition; reduction of GBA-related GSLs		GCase activation	TORC1 inhibition	GCase activation	Gene therapy, AAV-based
Status	Completed	Recruiting, estimated primary completion 2021	Completed	Ongoing; data expected 2020	Recruiting in Leiden (NL)	Clinical centers initiated
Phase	2		2a	1b/2a	1b	1b
Design	Multicenter, randomized, double-blind, placebo–controlled, sequential cohort	Prospective, single-center, open-label	Single-center open-label noncontrolled clinical	Multicenter, 2:1 randomized, double-blind, placebo-controlled	Randomized, placebo-controlled, double-blind, parallel study	Randomized, double-blind, sham procedure-controlled, ascending dose study
Total N of part.	17		8 + 10	45	Approximately 40	30/16
*GBA*-PD	√		√	√	√	√
Idiopathic PD	No		√	√	No	√
Age	18–80 yrs. (mean 58 years)		40–80, mean 60 years		18 years or older	
Duration	36 weeks	52 weeks + 104 weeks extension	6 months	4 weeks	28 days	
Doses tested	3 escalating doses	1 dose	Escalating oral dose to 1.26 g per day [420 mg 3 times per day]	300 mg; ± sirolimus	10 or 60 mg once daily	Two escalating dose cohorts

^a^See https://www.trialregister.nl/trial/7061 for more information

#### Treatment Directed at Modulation of Glycosphingolipid Turnover

Substrate reduction therapy inhibits the biosynthesis of lipid substrates and thereby prevents their accumulation. While this approach does not target the mutant gene or dysfunctional enzyme itself, it is an effective FDA-approved treatment of the systemic symptoms of Gaucher disease. However, the approved inhibitors show no effective CNS concentration and do not affect the neurological symptoms of Gaucher disease (i.e., Gaucher type 2 and 3). New glucosylceramide synthase inhibitors show good brain penetration, improved α-syn processing, and behavioral outcomes in mouse models [43, 64]. Based on these findings, Sanofi launched the MOVES-PD study, a randomized, double-blind, placebo-controlled trial, in order to assess the efficacy and safety of the glucosylceramide synthase inhibitor Venglustat (GZ/SAR402671). Initial results from a phase I study were recently published [65]. Briefly, 17 *GBA*-PD were enrolled (13 on Venglustat, 4 on placebo; mean age 58 years, mean disease duration 7 years) into this 36-week randomized, placebo-controlled, double-blind, sequential cohort study of once-daily Venglustat at three escalating doses. No serious adverse events occurred. Side effects included psychological, neurological, and gastrointestinal-related symptoms. Plasma and cerebrospinal fluid (CSF) glucosylceramide levels decreased in a dose-dependent manner (up to 75%), confirming target engagement. This favorable safety and tolerability profile of Venglustat at all doses led the company to advance to a phase II study (a 52-week trial which is currently ongoing) [65].

#### Treatment Directed at Restoration of Enzyme Function

Other therapies focus on the restoration of enzyme function, thus increasing glucocerebrosidase activity, especially in the brain. This can be achieved by [3)] ERT (enzyme replacement therapy), [9)] gene therapy, or [7)] small molecules. ERT is currently available for patients with Gaucher disease. However, ERT is based on large molecules and we are not familiar with any clinical trials of ERT that may penetrate the blood–brain barrier. Furthermore, there are no data on the use of ERT in PD.

Gene therapy uses adeno-associated virus vectors to deliver engineered DNA to human cells [43, 66]. For *GBA*, preclinical studies in mice demonstrated that adeno-associated virus-mediated expression of glucocerebrosidase corrected the aberrant accumulation of the toxic lipid glucosylsphingosine and reduced the levels of ubiquitin, tau, and α-syn aggregates [67]. Prevail Therapeutics, a new company launched in 2017, started a gene therapy clinical trial of PR001 in 16 *GBA-*PD patients in late 2019 (https://ir.prevailtherapeutics.com/news-releases/news-release-details/prevail-therapeutics-provides-program-update-pr001-parkinsons-0). The company will also test the compound in children with neuropathic Gaucher disease starting in the first half of 2020.

Furthermore, small molecules have also attracted attention [68]. Early glucocerebrosidase chaperones that underwent clinical trials for Gaucher disease included isofagomine (afegostat-tartrate, AT2101). This treatment did not lead to significant clinical improvement and further clinical development for this indication was discontinued [43]. Ambroxol, which is a promising small molecule chaperone widely used in Europe as a mucolytic agent, may potentially facilitate the transit of the misfolded GCase protein to the lysosome [69]. Its stabilizing and enhancing effects on the levels of GCase were identified in a high-throughput screen in the context of Gaucher disease [69]. It is pH dependent, binds to the active site of the GCase protein, and reduces GCase activity. Ambroxol has also been shown to improve lysosomal function and increase enzyme activity in in vitro studies utilizing dermal fibroblasts with *GBA* mutations (i.e. [70] as well as in studies performed on non-human primates (i.e. cynomolgus monkeys) with *GBA* mutations [71]). The effects of Ambroxol at high doses were recently tested in a single-center, open-label, non-controlled, clinical trial (escalating oral dose to 1.26 g per day [420 mg 3 times per day] which is considerably higher compared to the 30–120 mg/day used for the treatment of respiratory disease) [72]. Eighteen patients with moderately severe PD (mean age 60 years) completed the study, eight with *GBA* mutations and ten mutation-negative. Ambroxol penetrated CSF, but led to a 19% reduction from the mean baseline CSF GCase activity at day 186. There was an increase of CSF α-synuclein which the authors interpreted as an increase of extracellular export of the protein from the brain parenchyma. The drug was well tolerated, and no serious adverse events were reported [72]. For other indications, Ambroxol has been studied in > 15,000 patients in more than 100 trials with an excellent safety record [73]. As mentioned above, GCase activity is also reduced in iPD patients’ brains (SN) [74], making the therapy potentially relevant for iPD. The effect of Ambroxol in non-*GBA* PD [75] and non-*GBA* PD dementia [73] will be better understood once results from the two ongoing studies become available. These studies include ten non-*GBA* PD and 75 PD dementia patients [73, 76] (treated at daily doses of 420 mg/day or 525–1050 mg/day, respectively).

The effects of LTI-291, an activator of the GCase enzyme and another small molecule therapy, were studied in a 1-month phase 1b trial conducted in the Netherlands, where the frequency of *GBA* mutations was reported to be around 15% [48]. Around 40 *GBA*-PD patients participated. There were no safety events and data showed a good dose-dependent brain penetration (personal communication). The company, Lysosomal Therapeutics, Inc. (LTI), is a Massachusetts-based biotech venture which plans to develop therapies for Gaucher disease and other lysosome-based neurodegenerative diseases.

Small molecules can also modify *GBA*-PD by modifying *GBA*-independent pathways. One such example is RTB101, which is an inhibitor of the target of rapamycin complex 1 (TORC1) [77]. Rapamycin reached public attention when Bloomberg magazine publicized it as a potential “forever pill” in 2015. Known for its immunosuppressant properties, rapamycin prolongs lifespan by 15–25% in various non-mammalian organisms even when given late in life; it has also been found to increase healthspan. A 5-year study in dogs is planning on testing the geroprotective effects of RTB101 in mammals [78]. *TORC1* plays a role in cell growth and aging and is the switch between fasting and feeding states [77]. Mutations in *TORC1* cause focal cortical dysplasia, an established cause of epilepsy. The role of mTORC1 in regulating autophagy may also have implications for neurodegenerative diseases. In preclinical models, TORC1 inhibition induces autophagy and prevents neuronal loss [79]. It improves motor function in multiple PD models including α-syn transgenic mice and MPTP models [80]. In oncology cell cultures, treatment with RTB101 reduced the levels of glucosylceramide, the main substrate of GCase. There is a current phase 1b/2a trial of RTB101 in combination with sirolimus that involves 45 PD patients with or without a *GBA* mutation. The study was initiated in early 2019; data is expected in 2020.

#### Deep Brain Stimulation for *GBA*-PD

Outcome data after DBS are available for around 30 *GBA*-PD patients, 60% of whom showed a favorable response, whereas 30% showed a poor response [46]. However, sample sizes remain small and genetic testing does not yet provide added prognostic value over what can already be obtained through careful clinical assessment when the patient is evaluated for DBS surgery [81].

#### Targeting the Mitochondrial Pathways—Treatment for *PRKN*/*PINK1*

The second major pathway implicated in PD pathogenesis is mitochondrial metabolism, which is susceptible to damage by oxidative stress. A prevailing hypothesis is that mutations in *PRKN* and *PINK1* cause PD due to mitochondrial dysfunction. Clinically, people with PRKN PD and PINK1 PD display an earlier-onset, are sensitive to levodopa, and are prone to motor fluctuations. And,  these patients have minimal cognitive decline, even after many years of disease [13].

Hence, antioxidative strategies targeting Parkin (encoded by *PRKN*) and *PINK1*-mediated mitophagy are another emerging therapeutic approach for new treatments of PD. Several small molecule drugs that enhance mitophagy are in preclinical development and exploratory biomarkers for mitochondrial dysfunction and mitophagy activation are being tested. However, there are many aspects that need to be considered such as selecting the most effective and feasible targets in the mitophagy pathway, potential side effects, and stratification of the ideal population (for recent review, see Miller and Muquit 2019 [82]).

It is important to note that several compounds targeting mitochondria have been systematically studied in PD, including coenzyme Q10 and creatine. However, these trials failed to demonstrate a disease-modifying benefit in patients with PD. Of these, coenzyme Q10 is currently revisited in a “smaller, smarter trial” where patients are recruited based on their genetic risk profile [83]. Hence, patients will be stratified according to their risk for mitochondrial dysfunction (i.e., biallelic or monoallelic mutation in *PRKN* or *PINK1* vs. patients in whom omics suggest little or minimal mitochondrial dysfunction).

#### Challenges for Precision Clinical Trials

Surprisingly, the major challenge towards precision medicine clinical trials for PD is not in the basic understanding of the genes and their function, but rather in identifying mutation carriers for clinical trials (Table 4). There are many ways studies are trying to overcome this difficulty. In the case of interventions targeting α-syn, the challenge in identifying carriers of point mutations or copy number variants has led to clinical trials targeting patients with sporadic PD. In the case of *GBA* and *LRRK2*, it is less clear whether interventions on these pathways would help patients with sporadic PD. Given the relative rarity of mutation carriers, *GBA* and *LRRK2* trials may include patients in different stages of PD, as opposed to other clinical trials of disease modification which recruit patients in early PD who are often medication naïve. The inclusion of patients at different stages of PD requires the development of more sophisticated outcome measures that are less sensitive to symptomatic, dopaminergic therapy than the standard motor examination, the Unified Parkinson’s Disease Rating Scale (UPDRS).

**Table 4:** Top four challenges and barriers to effective clinical trials as perceived by health professionals, patients, and their caregivers—for the full list, see Mathur et al. (2015) [84]

Scientists and other health professionals	Patients and caregivers
Lack of funding	Risk of potential adverse consequences and potential side effects
Lack of administrative support and time available to manage the trial	Disruption to normal medication regimen
Slow and difficult recruitment of people	Prospect of receiving a placebo instead of the active drug
Lack of practical support	Upheaval and inconvenience to life that trial participation would cause

The feasibility of genetically-targeted clinical trials would require a significant change in the way genotyping is currently practiced in PD. Only a small fraction of PD patients are clinically genotyped in CLIA-approved laboratories and know their genetic status [85]. A few pharmaceutical companies launched major genotyping efforts, e.g., Denali Therapeutics in collaboration with Centogene (https://www.centogene.com/company/article/centogene-and-denali-therapeutics-announce-strategic-collaboration-to-recruit-lrrk2-patients-for-cli.html [online]). In this context, the Parkinson’s Foundation launched the PD GENEration initiative (currently conducted at 6 sites in the USA), which offers free genetic testing for clinically relevant PD-related genes and free genetic counseling to help participants better understand their results. More about current efforts in “Overcoming barriers to PD trial participation” are discussed in chapter 15 of this special issue.

Another major challenge is the identification of reliable and validated disease markers and outcome measurements that are suitable for targeted intervention. Past trials largely relied on clinical motor impressions (e.g. UPDRS in the “off” state during clinic visits) which may not robustly detect disease-modifying effects. Better biomarkers are needed to evaluate the effectiveness of treatments by reflecting drug target engagement, disease severity, and progression of disease. Having a reliable marker would enhance drug development. For example, in the case of Huntington’s disease, trials now include femtomolar-sensitive quantification (single molecule counting immunoassay) of the mutant protein in CSF [86]. For PD, Real-Time Quaking-Induced Conversion (RT-QuIC) techniques are being tested to determine their sensitivity and specificity [87]. There is also still a lack of reliable α-syn imaging methods which is a barrier to testing promising compounds; however, targeting a-syn is a major pursuit on the research front [88, 89]. Using artificial intelligence may advance the field further by identifying new biomarkers or facilitating biomarker validation and application.

Furthermore, better understanding of disease mechanisms linking these genes to PD is necessary. In *LRRK2* and *SNCA*, while detailed mechanisms are not fully understood, treatments are focused on reducing activity or expression. In the case of *GBA*, as outlined above, enhancing or restoring enzyme function are currently favored approaches. Drug development for other genetic forms lags behind (e.g., *PRKN*), because suitable druggable targets have not been identified. Concerted efforts are being made to identify a variety of disease progression markers including biospecimen-based (i.e., blood, urine, CSF or biopsy; molecular, genomic, cellular), clinical, imaging, or others (e.g., electrophyiological, behavioral, and environmental). Among these, the PPMI initiative is a valuable source that brings together longitudinal data and specimen collection from more than 1200 volunteers with PD [15].

#### Summary and Final Remarks

Recent failures in large Phase III clinical trials for PD suggest that disease modification will not succeed as long as PD is treated as a single disease with one pathophysiology. Therefore, we believe that precision medicine in PD may be a promising alternative and a timely approach.

As summarized in this review, several gene-based therapies are being tested in clinical trials and numerous more are in the pipeline. These are exciting times. However, the process of bringing a drug into the clinic is cumbersome [90]. Pharmaceutical Research and Manufacturers of America (PhRMA) estimate that for every 5000 to 10,000 compounds screened, only five enter human clinical trials, only one is approved by the Food and Drug Administration, and only two in ten drugs generate enough revenue to recoup their research and development costs [91]. Thus, setbacks are expected.

One unanswered question is who will benefit from new “precision” drugs. Will these be useful only for mutation carriers (and therefore require an orphan drug assignment) or will they be beneficial for the larger group of idiopathic PD or atypical parkinsonian disorders? It seems unlikely that these broad groups will all respond to the same drug unless there are shared downstream disease mechanisms. One may even have to differentiate among mutations within *GBA* due to the effect a specific mutation has on the protein. For example, the affinity of chaperones to a mutated enzyme may be different depending on the mutation. Furthermore, a new drug that facilitates protein function may fail in patients with null mutations who do not express any protein. The term “super precision medicine” has been used to capture this phenomenon. To be able to make the most out of clinical trial data, future clinical trials will benefit from pretrial genetic adjustment or, at the minimum, post-trial adjustment, and analysis for failed trials [92].

However, in addition to addressing the purely scientific aspects, success in drug development also depends on funding opportunities for late preclinical development phases which are significantly more expensive than early-stage discovery. Experience shows that promising ideas have often failed in this “Valley of Death” (a term coined by former NIH director, Elias Zerhouni) [93]. Feasible regulatory frameworks and efficient data-sharing ecosystems [94] that also ensure ethical leadership and governance [95] as well as new funding instruments (for example, similar to the recently initated public-private partnership set up to boost research and drug development in infectious diseases and thereby to address market failures [84]) will facilitate drug development.

Raising awareness and educating the community, including physicians, patients, and caregivers, will be an important step towards successful future studies [85]**.** Advancing precision medicine will not only shift current reactive approaches to proactive detection and prevention, but will also help the next generation of scientists develop creative new approaches for detecting, measuring, and treating Parkinson’s disease.

## Electronic Supplementary Material

ESM 1 (PDF 458 kb)

## References

[1] Global, regional, and national burden of Parkinson's disease (2018). 1990-2016: a systematic analysis for the Global Burden of Disease Study 2016. The Lancet Neurology.

[2] Abrahams E, President | Personalized Medicine Coalition Personalized Medicine: The Changing Landscape of Health Care; Key Note lecture, The 2nd Biomarker Meeting in Personalized Reproductive Medicine Valencia, Spain [www.comtecmed.com/biomarker/2014/Uploads/Editor/PDF/ppt/Edward%20Abrahams_Key%20Note%20Lecture.pdf]2014.

[3] Juengst E, McGowan ML, Fishman JR, Settersten RA (2016). From "Personalized" to "Precision" Medicine: The Ethical and Social Implications of Rhetorical Reform in Genomic Medicine. Hastings Cent Rep.

[4] Manolio TA, Collins FS, Cox NJ (2009). Finding the missing heritability of complex diseases. Nature.

[5] Gasser T (2015). Usefulness of Genetic Testing in PD and PD Trials: A Balanced Review. J Park Dis.

[6] Nalls MA, Blauwendraat C, Vallerga CL (2019). Identification of novel risk loci, causal insights, and heritable risk for Parkinson's disease: a meta-analysis of genome-wide association studies. The Lancet Neurology.

[7] Bandres-Ciga S, Diez-Fairen M, Kim JJ, Singleton AB (2020). Genetics of Parkinson's disease: An introspection of its journey towards precision medicine. Neurobiology of disease.

[8] Chang D, Nalls MA, Hallgrimsdottir IB (2017). A meta-analysis of genome-wide association studies identifies 17 new Parkinson's disease risk loci. Nature genetics.

[9] Fujita KA, Ostaszewski M, Matsuoka Y (2014). Integrating pathways of Parkinson's disease in a molecular interaction map. Mol Neurobiol.

[10] Kim J, Hu C, Moufawad El Achkar C (2019). Patient-Customized Oligonucleotide Therapy for a Rare Genetic Disease. The New England journal of medicine.

[11] Qing L (2020). Nusinersen as a therapeutic agent for spinal muscular atrophy. Yonsei Med J.

[12] Tabrizi SJ, Leavitt BR, Landwehrmeyer GB (2019). Targeting Huntingtin Expression in Patients with Huntington's Disease. The New England journal of medicine.

[13] Singleton A, Hardy J (2019). Progress in the genetic analysis of Parkinson's disease. Human molecular genetics.

[14] Romero K, Conrado D, Burton J (2019). Molecular Neuroimaging of the Dopamine Transporter as a Patient Enrichment Biomarker for Clinical Trials for Early Parkinson's Disease. Clin Transl Sci.

[15] Marek K, Chowdhury S, Siderowf A (2018). The Parkinson's progression markers initiative (PPMI) - establishing a PD biomarker cohort. Ann Clin Transl Neurol.

[16] Polymeropoulos MH, Lavedan C, Leroy E (1997). Mutation in the alpha-synuclein gene identified in families with Parkinson's disease. Science.

[17] Kruger R, Kuhn W, Muller T (1998). Ala30Pro mutation in the gene encoding alpha-synuclein in Parkinson's disease. Nat Genet.

[18] Zarranz JJ, Alegre J, Gomez-Esteban JC (2004). The new mutation, E46K, of alpha-synuclein causes Parkinson and Lewy body dementia. Ann Neurol.

[19] Appel-Cresswell S, Vilarino-Guell C, Encarnacion M (2013). Alpha-synuclein p.H50Q, a novel pathogenic mutation for Parkinson's disease. Mov Disord.

[20] Lesage S, Anheim M, Letournel F (2013). G51D alpha-synuclein mutation causes a novel parkinsonian-pyramidal syndrome. Annals of neurology.

[21] MDSGene Websitte wmo. Accessed Aug 8, 2019.

[22] Schenk DB, Koller M, Ness DK (2017). First-in-human assessment of PRX002, an anti-alpha-synuclein monoclonal antibody, in healthy volunteers. Movement disorders : official journal of the Movement Disorder Society.

[23] Jankovic J, Goodman I, Safirstein B (2018). Safety and Tolerability of Multiple Ascending Doses of PRX002/RG7935, an Anti-alpha-Synuclein Monoclonal Antibody, in Patients With Parkinson Disease: A Randomized Clinical Trial. JAMA Neurol.

[24] Weihofen A, Liu Y, Arndt JW (2019). Development of an aggregate-selective, human-derived alpha-synuclein antibody BIIB054 that ameliorates disease phenotypes in Parkinson's disease models. Neurobiology of disease.

[25] Schofield DJ, Irving L, Calo L (2019). Preclinical development of a high affinity alpha-synuclein antibody, MEDI1341, that can enter the brain, sequester extracellular alpha-synuclein and attenuate alpha-synuclein spreading in vivo. Neurobiology of disease.

[26] Price DL, Koike MA, Khan A (2018). The small molecule alpha-synuclein misfolding inhibitor, NPT200-11, produces multiple benefits in an animal model of Parkinson's disease. Sci Rep.

[27] Chatterjee D, Bhatt M, Butler D (2018). Proteasome-targeted nanobodies alleviate pathology and functional decline in an alpha-synuclein-based Parkinson's disease model. NPJ Parkinsons Dis.

[28] Kantor B, Tagliafierro L, Gu J (2018). Downregulation of SNCA Expression by Targeted Editing of DNA Methylation: A Potential Strategy for Precision Therapy in PD. Mol Ther.

[29] Heras-Garvin A, Stefanova N. MSA: From basic mechanisms to experimental therapeutics. Parkinsonism & related disorders 2020. 10.1016/j.parkreldis.2020.01.010 32005598

[30] Healy DG, Falchi M, O'Sullivan SS (2008). Phenotype, genotype, and worldwide genetic penetrance of LRRK2-associated Parkinson's disease: a case-control study. Lancet neurol.

[31] Lim SY, Tan AH, Ahmad-Annuar A, et al. Parkinson's disease in the Western Pacific Region. The Lancet Neurology 2019. 10.1016/S1474-4422(19)30195-4 31175000

[32] Ozelius LJ, Senthil G, Saunders-Pullman R (2006). LRRK2 G2019S as a cause of Parkinson's disease in Ashkenazi Jews. N Engl J Med.

[33] Jaleel M, Nichols RJ, Deak M (2007). LRRK2 phosphorylates moesin at threonine-558: characterization of how Parkinson's disease mutants affect kinase activity. The Biochemical journal.

[34] Atashrazm F, Dzamko N (2016). LRRK2 inhibitors and their potential in the treatment of Parkinson's disease: current perspectives. Clin Pharmacol.

[35] Blauwendraat C, Reed X, Kia DA (2018). Frequency of Loss of Function Variants in LRRK2 in Parkinson Disease. JAMA Neurol.

[36] Fuji RN, Flagella M, Baca M (2015). Effect of selective LRRK2 kinase inhibition on nonhuman primate lung. Sci Transl Med.

[37] Baptista MAS, Kalpana M; Barrett, T.; Bryce, D.K.; Ellis, M.; Estrada, A.A.; Fell, M.J.; Fiske, B.K.; Fuji, R.N.; et al.. LRRK2 kinase inhibitors induce a reversible effect in the lungs of non-human primates with no measurable pulmonary deficits. bioRxiv, 390815 2018.

[38] Whiffin NA, Irina M; Kleinman, A.; Marshall, J.L.; Minikel, E.V.; Goodrich, J.K.; Quaife, N.; Cole, J.B.; et al. Human loss-of-function variants suggest that partial LRRK2 inhibition is a safe therapeutic strategy for Parkinson’s disease. bioRxiv 2019; 10.1101/561472.

[39] Domingos S, Duarte T, Saraiva L, Guedes RC, Moreira R (2019). Targeting leucine-rich repeat kinase 2 (LRRK2) for the treatment of Parkinson's disease. Future Med Chem.

[40] Coutinho MF, Matos L, Santos JI, Alves S (2019). RNA Therapeutics: How Far Have We Gone?. Adv Exp Med Biol.

[41] Platt FM, d'Azzo A, Davidson BL, Neufeld EF, Tifft CJ (2018). Lysosomal storage diseases. Nat Rev Dis Primers.

[42] Zhao HT, John N, Delic V (2017). LRRK2 Antisense Oligonucleotides Ameliorate alpha-Synuclein Inclusion Formation in a Parkinson's Disease Mouse Model. Mol Ther Nucleic Acids.

[43] Sardi SP, Cedarbaum JM, Brundin P (2018). Targeted Therapies for Parkinson's Disease: From Genetics to the Clinic. Movement disorders : official journal of the Movement Disorder Society.

[44] Foroud T, Smith D, Jackson J (2015). Novel recruitment strategy to enrich for LRRK2 mutation carriers. Mol Genet Genomic Med.

[45] https://www.centogene.com/company/article/centogene-and-denali-therapeutics-announce-strategic-collaboration-to-recruit-lrrk2-patients-for-cli.html, accessed: 24th July, 2019 [online].

[46] Kuusimaki T, Korpela J, Pekkonen E, Martikainen MH, Antonini A, Kaasinen V. Deep brain stimulation for monogenic Parkinson's disease: a systematic review. Journal of Neurology 2019. 10.1007/s00415-019-09181-8 PMC7109183 30659355

[47] Gan-Or Z, Giladi N, Rozovski U (2008). Genotype-phenotype correlations between GBA mutations and Parkinson disease risk and onset. Neurology.

[48] den Heijer JM, Cullen VC, Quadri M, et al. A Large-Scale Full GBA1 Gene Screening in Parkinson's Disease in the Netherlands. Mov Disord 2020. 10.1002/mds.28112 PMC7540512 32618053

[49] Zuckerman S, Lahad A, Shmueli A (2007). Carrier screening for Gaucher disease: lessons for low-penetrance, treatable diseases. JAMA.

[50] Hruska KS, LaMarca ME, Scott CR, Sidransky E (2008). Gaucher disease: mutation and polymorphism spectrum in the glucocerebrosidase gene (GBA). Hum Mutat.

[51] Sidransky E, Nalls MA, Aasly JO (2009). Multicenter analysis of glucocerebrosidase mutations in Parkinson's disease. N Engl J Med.

[52] Roshan Lal T, Sidransky E. The Spectrum of Neurological Manifestations Associated with Gaucher Disease. Diseases 2017;5. 10.3390/diseases5010010 PMC5456331 28933363

[53] Rana HQ, Balwani M, Bier L, Alcalay RN (2013). Age-specific Parkinson disease risk in GBA mutation carriers: information for genetic counseling. Genetics in medicine : official journal of the American College of Medical Genetics.

[54] Clark LN, Ross BM, Wang Y (2007). Mutations in the glucocerebrosidase gene are associated with early-onset Parkinson disease. Neurology.

[55] Clark LN, Kartsaklis LA, Wolf Gilbert R (2009). Association of glucocerebrosidase mutations with dementia with lewy bodies. Arch Neurol.

[56] Geiger JT, Ding J, Crain B (2016). Next-generation sequencing reveals substantial genetic contribution to dementia with Lewy bodies. Neurobiol dis.

[57] Mitsui J, Matsukawa T, Sasaki H (2015). Variants associated with Gaucher disease in multiple system atrophy. Ann clin transl neurol.

[58] Sklerov MK,Un J; Liong, C.; Marder, K.; Pauciulo, M.; Nichols, W.C.; Chung, W.K.; Honig, L.S.; Cortes, E.; Vonsattel, J.P.. Frequency of GBA Variants in Autopsy-proven Multiple System Atrophy. Mov Disord Clin Pract 2017. 10.1002/mdc3.12481 PMC5614491 28966932

[59] Segarane B, Li A, Paudel R (2009). Glucocerebrosidase mutations in 108 neuropathologically confirmed cases of multiple system atrophy. Neurology.

[60] Nishioka K, Ross OA, Vilarino-Guell C (2011). Glucocerebrosidase mutations in diffuse Lewy body disease. Parkinsonism Relat Disord.

[61] Goker-Alpan O, Giasson BI, Eblan MJ (2006). Glucocerebrosidase mutations are an important risk factor for Lewy body disorders. Neurology.

[62] Mazzulli JR, Xu YH, Sun Y (2011). Gaucher disease glucocerebrosidase and alpha-synuclein form a bidirectional pathogenic loop in synucleinopathies. Cell.

[63] Barkhuizen M, Anderson DG, Grobler AF (2016). Advances in GBA-associated Parkinson's disease--Pathology, presentation and therapies. Neurochem Int.

[64] Sardi SP, Viel C, Clarke J (2017). Glucosylceramide synthase inhibition alleviates aberrations in synucleinopathy models. Proceedings of the National Academy of Sciences of the United States of America.

[65] Peterschmitt MJ, Gasser T, Isaacson S, Kulisevsky J, Mir P, Simuni T, Wills AM, Guedes LC (2019). Safety, tolerability and pharmacokinetics of oral venglustat in Parkinson disease patients with a GBA mutation. Mol genet metabol rep.

[66] Hitti FL, Yang AI, Gonzalez-Alegre P, Baltuch GH. Human gene therapy approaches for the treatment of Parkinson's disease: An overview of current and completed clinical trials. Parkinsonism & Related Disorders 2019. 10.1016/j.parkreldis.2019.07.018 31324556

[67] Sardi SP, Clarke J, Viel C (2013). Augmenting CNS glucocerebrosidase activity as a therapeutic strategy for parkinsonism and other Gaucher-related synucleinopathies. Proceedings of the National Academy of Sciences of the United States of America.

[68] McMahon B, Aflaki E, Sidransky E (2016). Chaperoning glucocerebrosidase: a therapeutic strategy for both Gaucher disease and Parkinsonism. Neural Regen Res.

[69] Maegawa GH, Tropak MB, Buttner JD (2009). Identification and characterization of ambroxol as an enzyme enhancement agent for Gaucher disease. The Journal of biological chemistry.

[70] McNeill A, Magalhaes J, Shen C (2014). Ambroxol improves lysosomal biochemistry in glucocerebrosidase mutation-linked Parkinson disease cells. Brain : a journal of neurology.

[71] Migdalska-Richards A, Ko WKD, Li Q, Bezard E, Schapira AHV. Oral ambroxol increases brain glucocerebrosidase activity in a nonhuman primate. Synapse 2017;71. 10.1002/syn.21967 PMC5485051 28295625

[72] Mullin S, Smith L, Lee K, et al. Ambroxol for the Treatment of Patients With Parkinson Disease With and Without Glucocerebrosidase Gene Mutations: A Nonrandomized, Noncontrolled Trial. JAMA Neurol 2020. 10.1001/jamaneurol.2019.4611 PMC6990847 31930374

[73] Silveira CRA, MacKinley J, Coleman K (2019). Ambroxol as a novel disease-modifying treatment for Parkinson's disease dementia: protocol for a single-centre, randomized, double-blind, placebo-controlled trial. BMC Neurol.

[74] Gegg ME, Burke D, Heales SJ (2012). Glucocerebrosidase deficiency in substantia nigra of parkinson disease brains. Annals of neurology.

[75] Trust hcginNsbSUaCPs.

[76] Institute hcginNsbSLHR.

[77] Saxton RA, Sabatini DM (2017). mTOR Signaling in Growth, Metabolism, and Disease. Cell.

[78] Savage N (2017). New tricks from old dogs join the fight against ageing. Nature.

[79] Spilman P, Podlutskaya N, Hart MJ (2010). Inhibition of mTOR by rapamycin abolishes cognitive deficits and reduces amyloid-beta levels in a mouse model of Alzheimer's disease. PLoS One.

[80] Decressac M, Bjorklund A (2013). mTOR inhibition alleviates L-DOPA-induced dyskinesia in parkinsonian rats. J Parkinsons Dis.

[81] Ligaard J, Sannaes J, Pihlstrom L (2019). Deep brain stimulation and genetic variability in Parkinson's disease: a review of the literature. NPJ Parkinsons Dis.

[82] Miller S, Muqit MMK (2019). Therapeutic approaches to enhance PINK1/Parkin mediated mitophagy for the treatment of Parkinson's disease. Neurosci Lett.

[83] e:Med. Mitochondrial endophenotypes of PD, MitoPD, clinical trial https://www.sys-med.de/en/demonstrators/mitopd/sp-6/ [online]. Accessed 20 Feb.

[84] Mathur S, DeWitte S, Robledo I, Isaacs T, Stamford J (2015). Rising to the Challenges of Clinical Trial Improvement in Parkinson's Disease. J Parkinsons Dis.

[85] Alcalay RN, Kehoe C, Shorr E (2020). Correction: Genetic testing for Parkinson disease: current practice, knowledge, and attitudes among US and Canadian movement disorders specialists. Genetics in Medicine: official journal of the American College of Medical Genetics.

[86] Zeun P, Scahill RI, Tabrizi SJ, Wild EJ (2019). Fluid and imaging biomarkers for Huntington's disease. Mol Cell Neurosci.

[87] van Rumund A, Green AJE, Fairfoul G, Esselink RAJ, Bloem BR, Verbeek MM (2019). alpha-Synuclein real-time quaking-induced conversion in the cerebrospinal fluid of uncertain cases of parkinsonism. Ann Neurol.

[88] Teil M, Arotcarena ML, Faggiani E, Laferriere F, Bezard E, Dehay B. Targeting alpha-synuclein for PD Therapeutics: A Pursuit on All Fronts. Biomolecules 2020;10. 10.3390/biom10030391 PMC7175302 32138193

[89] Merchant KM, Cedarbaum JM, Brundin P (2019). A Proposed Roadmap for Parkinson's Disease Proof of Concept Clinical Trials Investigating Compounds Targeting Alpha-Synuclein. J Parkinsons Dis.

[90] Long G. The Biopharmaceutical Pipeline: Innovative Therapies in Clinical Development. Analysis Group https://www.analysisgroupcom/uploadedfiles/content/insights/publishing/the_biopharmaceutical_pipeline_report_2017pdf 2017.

[91] (PhRMA) PRaMoA. PhRMA SPECIAL 301 SUBMISSION 2009 OVERVIEW. 2009;Special 301 Submission.

[92] Leonard H, Blauwendraat C, Krohn L, et al. Genetic variability and potential effects on clinical trial outcomes: perspectives in Parkinson's disease. Journal of Medical Genetics 2019. 10.1136/jmedgenet-2019-106283 PMC8474559 31784483

[93] Ghoda LY, Rosen ST, Kwak LW. The changing investment in translational science by academic medical centers: HOPE in the Valley of Death. J Clin Invest 2020. 10.1172/JCI138640 PMC7324205 32484455

[94] Blasimme A, Fadda M, Schneider M, Vayena E (2018). Data Sharing For Precision Medicine: Policy Lessons And Future Directions. Health Aff (Millwood).

[95] Ho CWLA, Ali J, Caals K. Ensuring Trustworthy Use of Artificial Intelligence and Big Data Analytics in Health Insurance. Bull World Health Organ 2020;98:263-269. 10.2471/BLT.19.234732 PMC7133481 32284650

